# Chromatin remodeler ALC1 prevents replication-fork collapse by slowing fork progression

**DOI:** 10.1371/journal.pone.0192421

**Published:** 2018-02-06

**Authors:** Masato Ooka, Takuya Abe, Kosai Cho, Kaoru Koike, Shunichi Takeda, Kouji Hirota

**Affiliations:** 1 Department of Chemistry, Graduate School of Science and Engineering, Tokyo Metropolitan University, 1–1 Minami-osawa, Hachioji, Tokyo, Japan; 2 Department of Radiation Genetics, Graduate School of Medicine, Kyoto University, Yoshidakonoe, Sakyo-ku, Kyoto, Japan; 3 Department of Primary Care and Emergency Medicine, Kyoto University Graduate School of Medicine, Sakyo-ku, Kyoto, Japan; University of South Alabama Mitchell Cancer Institute, UNITED STATES

## Abstract

ALC1 (amplified in liver cancer 1), an SNF2 superfamily chromatin-remodeling factor also known as CHD1L (chromodomain helicase/ATPase DNA binding protein 1-like), is implicated in base-excision repair, where PARP (Poly(ADP-ribose) polymerase) mediated Poly(ADP-ribose) signaling facilitates the recruitment of this protein to damage sites. We here demonstrate the critical role played by ALC1 in the regulation of replication-fork progression in cleaved template strands. To analyze the role played by ALC1 as well as its functional relationship with PARP1, we generated *ALC1*^*-/-*^, *PARP1*^*-/-*^, and *ALC1*^*-/-*^*/PARP1*^*-/-*^ cells from chicken DT40 cells. We then exposed these cells to camptothecin (CPT), a topoisomerase I poison that generates single-strand breaks and causes the collapse of replication forks. The *ALC1*^*-/-*^ and *PARP1*^*-/-*^ cells exhibited both higher sensitivity to CPT and an increased number of chromosome aberrations, compared with *wild-type* cells. Moreover, phenotypes were very similar across all three mutants, indicating that the role played by ALC1 in CPT tolerance is dependent upon the PARP pathway. Remarkably, inactivation of ALC1 resulted in a failure to slow replication-fork progression after CPT exposure, indicating that ALC1 regulates replication-fork progression at DNA-damage sites. We disrupted ATPase activity by inserting the E165Q mutation into the *ALC1* gene, and found that the resulting *ALC1*^*-/E165Q*^ cells displayed a CPT sensitivity indistinguishable from that of the null-mutant cells. This observation suggests that ALC1 contributes to cellular tolerance to CPT, possibly as a chromatin remodeler. This idea is supported by the fact that CPT exposure induced chromatin relaxation in the vicinity of newly synthesized DNA in *wild-type* but not in *ALC1*^*-/-*^ cells. This implies a previously unappreciated role for ALC1 in DNA replication, in which ALC1 may regulate replication-fork slowing at CPT-induced DNA-damage sites.

## Introduction

ALC1 is a member of the SNF2 superfamily of ATPases, which can function as chromatin-remodeling enzymes [1–3]. ALC1 possesses a macrodomain that recognizes Poly(ADP-ribose) (PAR) *in vitro* and *in vivo* [4, 5]. PAR is generated by Poly(ADP-ribose) polymerase (PARP), which is activated by single-strand DNA breaks (SSBs) and gaps [6, 7]. SSBs occur during base-excision repair (BER) [8], which removes base damage, including oxidation and alkylation of bases [8, 9]. Formation of PAR at chromatin proteins in the vicinity of SSBs facilitates the recruitment of ALC1 and other BER factors to damaged-base sites [4, 10]. PARP1 stimulates the chromatin-repositioning enzyme activity of ALC1 [11, 12]. Collaboration between ALC1 and PARP is also implicated in the nucleotide-excision repair of ultraviolet light (UV)-induced DNA damage [13]. It remains unclear whether ALC1 plays a role in DNA replication.

Camptothecins (CPTs) have emerged as a promising chemotherapeutic agent for cancer [14, 15]. These drugs target topoisomerase I (TOPI), which mediates DNA nicking, rotation, and resealing associated with DNA relaxation during replication [14, 15]. CPT interferes with TOPI after DNA nicking, resulting in a covalently attached TOPI-DNA cleavage complex (TOPI-cc) at the 3’ SSB end. DNA double-strand breaks (DSBs) are created by the collision of replication forks with TOPI-cc [16–19], which explains why rapidly cycling cells exhibit a strong sensitivity to CPT [14, 15]. PARP1 is able to interact and play role with Tyrosyl-DNA phosphodiesterase 1 (TDP1), which eliminates TOP1 from TOPI-cc [20]. Although this decreases the number of collisions between TOPI-ccs and replication forks, PARP1 decreases the rate of replication-fork progression upon exposure of cells to CPT [21, 22]. Thus, PARP1 may decrease the progression of replication forks independently of the activation of TDP1 by PARP, which would further reduce the number of collisions between TOP1-ccs and replication forks. Thus, PARP1 contributes to cellular tolerance to CPT via two independent mechanisms: the activation of TDP1 and the regulation of replication-fork progression following DNA damage. It is unclear whether ALC1 collaborates with PARP1 in the latter mechanism.

Using a *PARP1*^*-/-*^ clone generated from the chicken DT40 B cell line is advantageous for analyzing the PARP pathway, as *PARP1*^*-/-*^ DT40 cells are equivalent to *PARP1*^*-/-*^*/PARP2*^*-/-*^ mammalian cells due to the absence of the *PARP2* gene in the chicken genome [23]. In this study, we explore the role played by ALC1 as well as its functional relationship with the PARylation pathway. *ALC1*^*-/-*^ cells were more sensitive to CPT than were *wild-type* cells, while loss of ALC1 had no detectable impact on the sensitivity of *PARP1*^*-/-*^ cells to CPT. This data establishes an epistatic relationship between *ALC1* and *PARP1* in cellular tolerance to CPT. Moreover, loss of ALC1 resulted in the failure of safe replication-fork arrest. In summary, our data suggest that ALC1 and PARP1 collaborate to mediate safe replication-fork arrest at CPT-induced TopI-cc sites. This study unveils the previously unappreciated role played by ALC1 in regulating replication-fork progression to prevent the collapse of replication forks at TopI-cc sites.

## Materials and methods

### DT40 cell culture, ALC1 targeting construct, and ALC1-ATPase dead knock-in construct

The DT40 cell line was obtained from the Takeda laboratory (Kyoto University) [24]. DT40 cells were cultured in RPMI-1640 medium supplemented with 50 μM β-mercaptoethanol, penicillin, streptomycin, 10% fetal calf serum, and 1% chicken serum (Gibco) at 39.5°C. Methods used to generate the ALC1-targeting construct and the ALC1-ATPase dead knock-in construct were as described previously [25].

### Generation of *ALC1*^*-/-*^ and *ALC1*^*-/-*^*/PARP1*^*-/-*^ mutant cells

We sequentially transfected ALC1-bsr and ALC1-hisD with targeting constructs to obtain *ALC1*^-/-^ cells from *wild-type* DT40 cells as described previously [25]. We similarly generated *ALC1*^*-/-*^*/PARP1*^*-/-*^ mutants from previously established *PARP1*^*-/-*^ DT40 cells [26].

### Measurement of sensitivity to genotoxic agents

Camptothecin (TopoGEN, Inc.), olaparib (AZD-2281, Astrazeneca), etoposide (VP16, Funakoshi), ICRF-193 (Funakoshi) and cis-diamminedichloroplatinum(II) (cisplatin, Nippon Kayaku) were used for the sensitivity assay, as described previously [27–30]. 10^4^ cells were plated in duplicate onto 24-well cluster plates containing 1m of complete medium supplemented with the above-mentioned reagents and further incubated for 48 h. 3 × 10^5^ cells were exposed to UV or irradiated by a ^137^Cs γ-ray source. 10^4^ cells were then plated in duplicate onto 24-well cluster plates containing 1m of complete medium and culture for 48 h. 100 μl of incubated cell were transferred to 96-well plates and measured the amount of ATP using CellTiter-Glo (Promega), according to the manufacturer's instructions. Luminescence was measured by Fluoroskan Ascent FL (Thermo Fisher Scientific Inc, Whaltham, MA)

### Chromosomal aberration analysis

DT40 clones were treated with 0.06% colcemid (Gibco BRL) for 2.5 h to arrest cells in the M-phase. Cells were pelleted by centrifugation, resuspended in 1 ml of 75 mM KCl for 15 min at room temperature, and fixed in 5 ml of a freshly prepared 3:1 mixture of methanol and acetic acid (Carnoy’s solution). The pelleted cells were then resuspended in 5 ml of Carnoy’s solution, dropped onto clean glass slides and air-dried. The slides were stained with a 5% HARLECO Giemsa stain solution (Nacalai Tesque) for 10 min, rinsed with water and acetone, and dried. All chromosomes in each mitotic cell were scored at 1000 × magnification.

### DNA fiber assay

The DNA fiber assay was performed as previously described [27, 28], with a slight modification of the labeling method for the replicated tract. Cells were sequentially labeled for 15 min with 25 μM CldU and for 15 min with 250 μM IdU. Fiber length was measured using Image J (https://imagej.nih.gov/ij/), and the CldU/IdU ratio was calculated. Measurements were recorded from areas of the slides with untangled DNA fibers to prevent the possibility of recording labeled patches from tangled bundles of fibers.

### Micrococcal nuclease digestion assay

5 ×10^7^ DT40 cells were pulse-labeled with 20 μM BrdU for 10 min. Cells were then harvested and washed and resuspended in medium either with CPT (20 μM) or without CPT and further cultured for 15 min. Partial digestion of chromatin DNA with Micrococcal nuclease (MNase) was performed as described previously [31–33], with slight modifications. Briefly, the above-mentioned BrdU-labeled cells were suspended in 0.5 ml of lysis buffer (18% Ficoll 400, 10 mM KH_2_PO_4_, 10 mM K_2_HPO_4_, 1 mM MgCl_2_, 0.25 mM EGTA, 0.25 mM EDTA, and 1 mM Pefabloc SC [Roche, Mannheim, Germany]). After centrifugation at 14,000 rpm for 30 min at 4°C, the crude chromatin fraction was resuspended in 0.3 ml of buffer A (10 mM Tris-HCl [pH 8.0], 150 mM NaCl, 5 mM KCl, and 1 mM EDTA) containing a proteinase inhibitor cocktail (Complete, Roche). After addition of CaCl_2_ (5 mM final concentration), 0.1 ml aliquots of crude chromatin suspension were digested with several different amounts of MNase (0, 5, 10, and 20 U/ml) at 37°C for 5 min. The reaction was terminated by adding 25 mM EDTA, and DNA was purified. DNA samples were resolved in 2% agarose gel electrophoresis followed by membrane transfer and immunodetection using anti-BrdU antibody (Roche).

## Results and discussion

### *ALC1*^*-/-*^ cells are moderately sensitive to camptothecin

**Fig 1:**
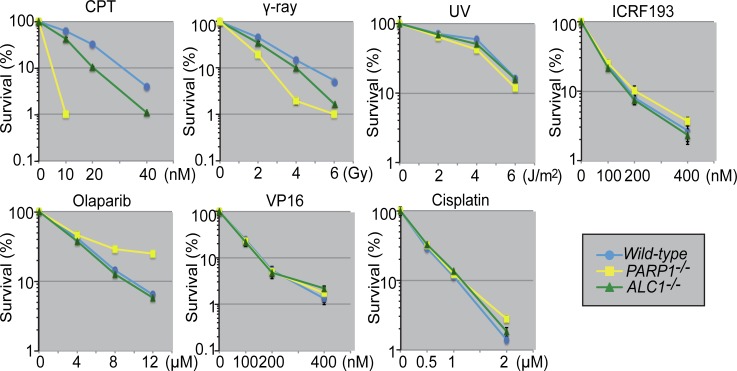
Role played by ALC1 in cell tolerance to CPT. Indicated cells were incubated in medium containing the following DNA-damaging agents at 39.5°C for 48 h: CPT (camptothecin, a topoisomerase 1 poison), γ-ray, UV, ICRF-193 (a catalytic topoisomerase 2 inhibitor), olaparib (a PARP inhibitor), VP16 (a topoisomerase 2 poison), and cisplatin (cis-diamminedichloroplatinum[II]). Then, cell viability was assessed by ATP assay as described in materials and methods. Dosage is displayed on the x-axis on a linear scale, while cell-survival percentage is displayed on the y-axis on a logarithmic scale. Error bars represent standard deviations from three independent experiments.

To determine in which DNA-repair pathways ALC1 plays a role, we generated *ALC1*^-/-^ cells from the chicken DT40 cell line and measured cell survival after exposure to a wide variety of DNA-damaging agents. *ALC1*^*-/-*^ and *wild-type* cells exhibited an indistinguishable sensitivity to cisplatin, UV, VP16, ICRF-193, and olaparib, but the *ALC1*^*-/-*^ cells were moderately more sensitive to CPT than *wild-type* cells and showed four-fold reduction of viability in comparison to *wild-type* cells (Fig 1, at 40 nM CPT, *wild-type* and *ALC1*^-/-^ cells showed 4% and 1% of survived cells, respectively). *PARP1*^*-/-*^ cells were critically more sensitive to CPT than *wild-type* and *ALC1*^*-/-*^ cells and showed 60-fold reduction of viability in comparison to *wild-type* cells (Fig 1, at 10 nM CPT, *wild-type* and *PARP1*^-/-^ cells showed 60% and 1% of survived cells, respectively). These data indicate that ALC1 is involved in the cellular tolerance to CPT. Given the role ALC1 plays in PARP-mediated DNA repair [4, 11–13, 25, 34], we suggest that the two repair factors may work along a common pathway.

### *ALC1* and *PARP1* have an epistatic relationship in cellular tolerance to CPT

**Fig 2:**
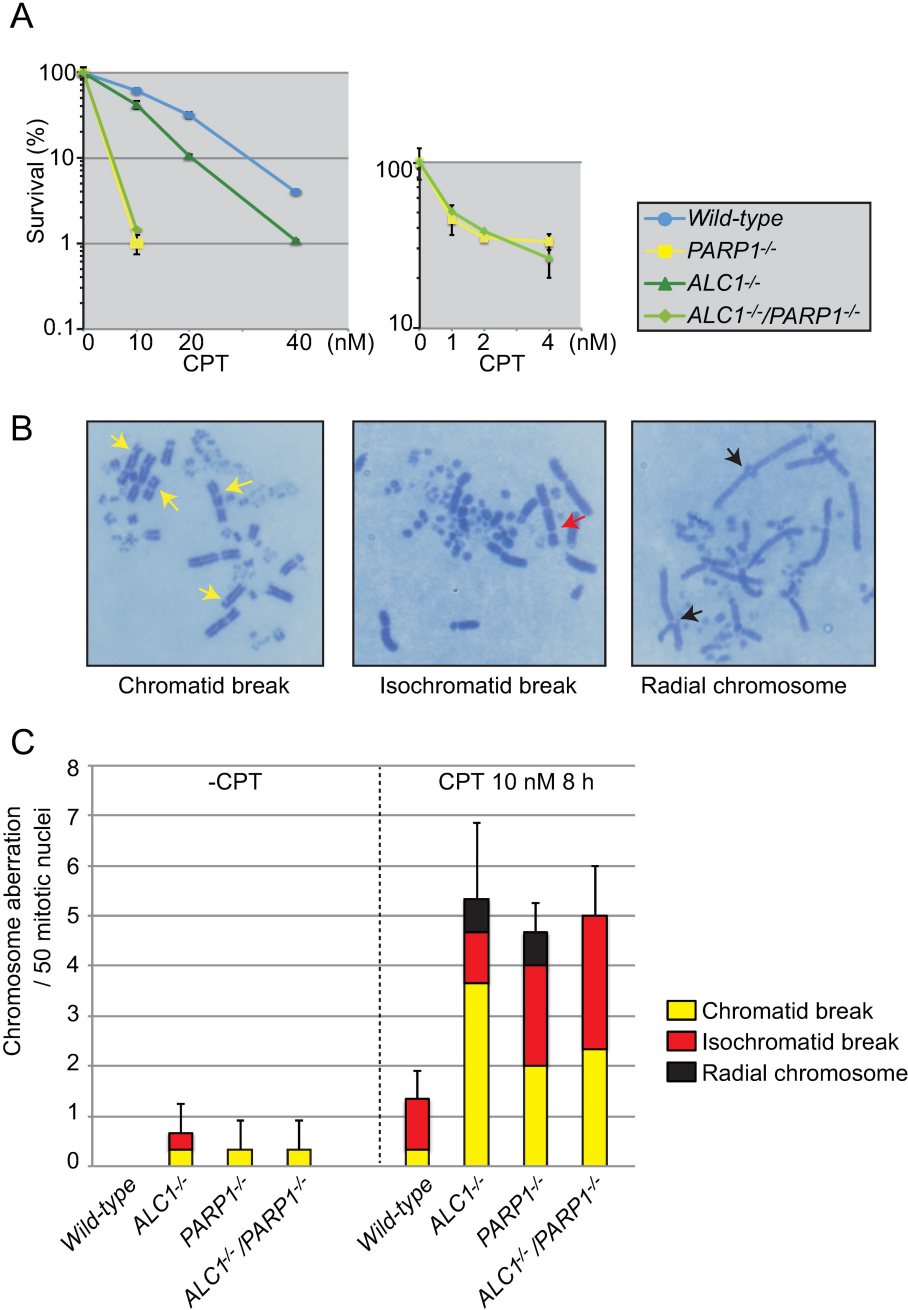
Epistatic relationship between *ALC1*^-/-^ and *PARP1*^-/-^ in cellular tolerance to CPT. (A) Cells with indicated genotypes were assessed for CPT sensitivity as in Fig 1. Dose is displayed on the x-axis, while the cell-survival percentage is displayed on y-axis. Error bars represent standard deviations from three independent experiments. The data for *wild-type*, *ALC1*^-/-^ and *PARP1*^-/-^ were same with the data in Fig 1. (B) Representative images showing DT40 chromosomes. Chromosomes from *wild-type* DT40 cells treated with mitomycinC were analyzed as described in Materials and Methods. The yellow, red and black arrows indicate chromatid breaks, isochromatid breaks and radial chromosomes, respectively. (C) Number of chromatid breaks, isochromatid breaks, and radial chromosomes in 50 mitotic cells. Chicken DT40 cells were exposed to CPT (10 nM) for 8 h with colcemid added 2.5 h before harvest to accumulate mitotic cells. Error bars represent standard deviations from three independent experiments.

We next compared *ALC1*^*-/-*^, *PARP1*^*-/-*^, and *ALC1*^*-/-*^*/PARP1*^*-/-*^ cells for sensitivity to CPT (Fig 2A). The *ALC1*^*-/-*^ cells were less sensitive than the *PARP1*^*-/-*^ cells, with the *PARP1*^*-/-*^ and *ALC1*^*-/-*^*/PARP1*^*-/-*^ cells exhibiting a similar sensitivity (Fig 2A). We therefore conclude that ALC1 and PARP1 collaborate in contributing to cellular tolerance to CPT. *ALC1*^*-/-*^, *PARP1*^*-/-*^, and *ALC1*^*-/-*^*/PARP1*^*-/-*^ cells all showed an increase in the number of chromosome breaks after exposure to CPT, with the *PARP1*^*-/-*^ and *ALC1*^*-/-*^*/PARP1*^*-/-*^ cells exhibiting a very similar number of chromosome aberrations (Fig 2B). Note that loss of PARP1 in *ALC1*^*-/-*^ cells changed the type of chromosome aberrations and resulted in an increase of isochromatid breaks (i.e., breaks at the same site of both sister chromatids.) This type of break is likely caused by defective resolution of recombination intermediates [30], and thus implying a possible involvement of PARP1 in the resolution of recombination intermediates.

### ALC1 induces chromatin relaxation around TOPI-cc

**Fig 3:**
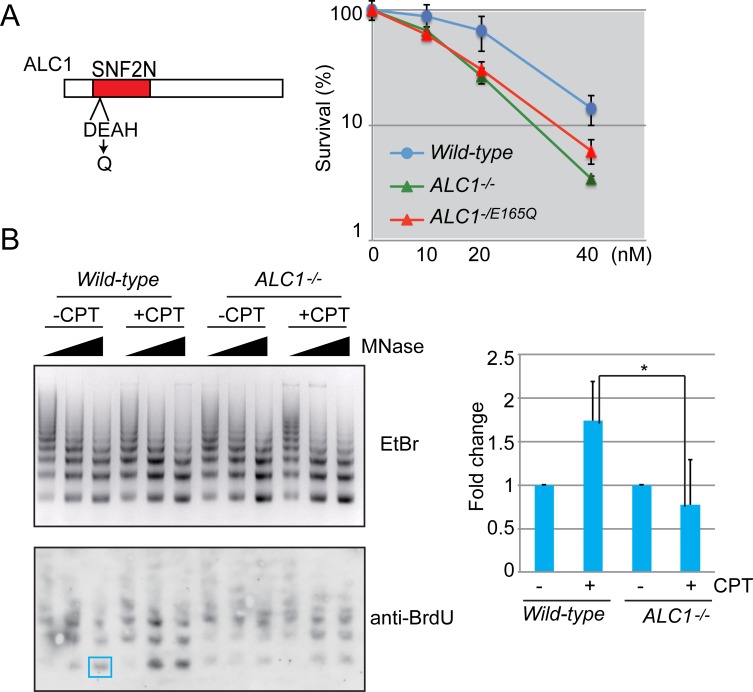
ALC1 induces chromatin relaxation around TOPI-cc. (A) (Left) Schematic representation of the ALC1 domain. Glu-165 on ALC1 was altered by Gln to inhibit the ATPase activity of ALC1. (Right) The ALC1-ATPase dead mutant (*ALC1*^*-/E165Q*^) and the *ALC1*^-/-^ cells showed a similar sensitivity to CPT. Error bars represent standard deviations from three independent experiments. (B) Nucleosome assembly in close proximity to TOPI-cc. 5 ×10^7^ *wild-type* and *ALC1*^-/-^ cells were pulse-labeled with BrdU for 10 min followed by treatment with CPT (20 μM) for 15 min. Nuclei were prepared and treated with 5, 10, and 20 units/ml MNase for 5 min. DNA was resolved in 2% agarose gel and stained with ethidium bromide (EtBr) (upper panel), transferred onto a membrane, and detected with an anti-BrdU antibody (lower panel). Blue box indicates quantified band corresponding to mononucleosome. *P*-value was calculated by Student’s *t*-test (*P<0.05).

To determine ALC1’s function as a chromatin-remodeler, we measured cellular sensitivity to CPT in *ALC1*^*-/E165Q*^ mutant cells, in which the ATPase activity of ALC1 is inactivated by mutating the essential E165 to Q (the same mutation used in previous biochemical studies [4, 11]) (Fig 3A). *ALC1*^*-/E165Q*^ and *ALC1*^*-/-*^ cells showed a virtually identical CPT sensitivity (Fig 3A), indicating that the chromatin-remodeling activity of ALC1 is required for cellular tolerance to CPT. This requirement suggests that ALC1 might induce chromatin relaxation around TOPI-cc and thereby participate in the CPT-tolerance mechanism. To examine this hypothesis, we analyzed the degree of chromatin condensation around replication forks stalled at TOPI-cc using an MNase digestion assay. We labeled newly replicated DNA with BrdU for 10 min, treated it with CPT for 15 min, then detected it with an anti-BrdU antibody (Fig 3B). The partially digested product corresponding to mononucleosomes (146 bp DNA, blue box in Fig 3B) was quantified. Following treatment with 20 μM CPT, MNase sensitivity was significantly increased and mononucleosomes were more efficiently digested in *wild-type* cells (Fig 3B), suggesting a change of chromatin state into a more open configuration. In marked contrast, MNase sensitivity in *ALC1*^*-/-*^ cells was slightly reduced by the CPT treatment, suggesting that chromatin relaxation around the TOPI-cc site cannot be maintained in the absence of ALC1. We thus conclude that CPT induces chromatin decondensation and that ALC1 might be required for this process. This conclusion is consistent with the recently demonstrated pivotal role played by ALC1 in chromatin remodeling at DNA damages induced by laser microirradiation [34].

### Distinct contribution of the ALC1-PARP1 and RNF8 pathways to CPT tolerance

**Fig 4:**
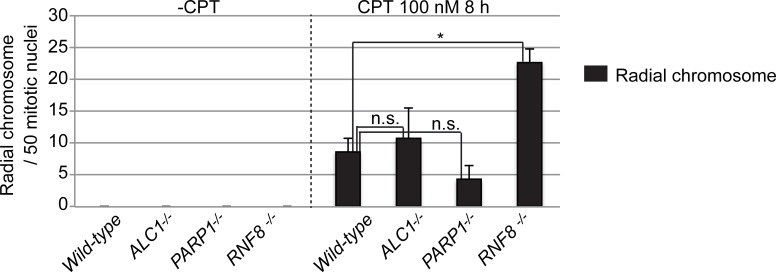
The ALC1-PARP1 axis is not required for the suppression of toxic NHEJ following CPT exposure. Number of radial chromosomes in 50 mitotic cells. DT40 cells were exposed to CPT (100 nM) for 8 h with colcemid added 2.5 h before harvest to accumulate mitotic cells. Error bars represent standard deviations from three independent experiments. *P*-value was calculated by a Student’s *t*-test (*P<0.05); n.s. = not significant.

*RNF8*^-/-^ cells showed a higher sensitivity to CPT than did *wild-type* cells [35], a phenotypic trait that is similar to that of *ALC1*^*-/-*^ and *PARP1*^*-/-*^. We demonstrated that RNF8 suppressed toxic non-homologous end-joining (NHEJ), as evidenced by the increased number of radial chromosomes in the *RNF8*^*-/-*^ cells [35]. This phenomenon occurs mainly as a consequence of aberrant NHEJ, as the number of radial chromosome events in NHEJ-deficient *KU70*^*-/-*^ cells is several times lower than in *wild-type* cells [35, 36]. To examine whether the collaborative ALC1-PARP pathway also contributes to CPT tolerance by suppressing toxic NHEJ, we measured the number of radial chromosomes. Strikingly, there was no increase in radial chromosomes in either *ALC1*^*-/-*^ or *PARP1*^*-/-*^, whereas the number of radial chromosome in *RNF8*^-/-^ was two times higher than in *wild-type* cells (Fig 4). We thus conclude that the ALC1-PARP pathway contributes to CPT tolerance by some other mechanism(s) than the suppression of toxic NHEJ. This conclusion is consistent with our previous study [26, 35], which showed that RNF8 and RAD18 collaboratively suppress toxic NHEJ following CPT damage [35], whilePARP1 contributes to cellular tolerance to CPT independently of RAD18 [26].

### Role played by ALC1 in the regulation of replication forks at CPT-induced lesions

**Fig 5:**
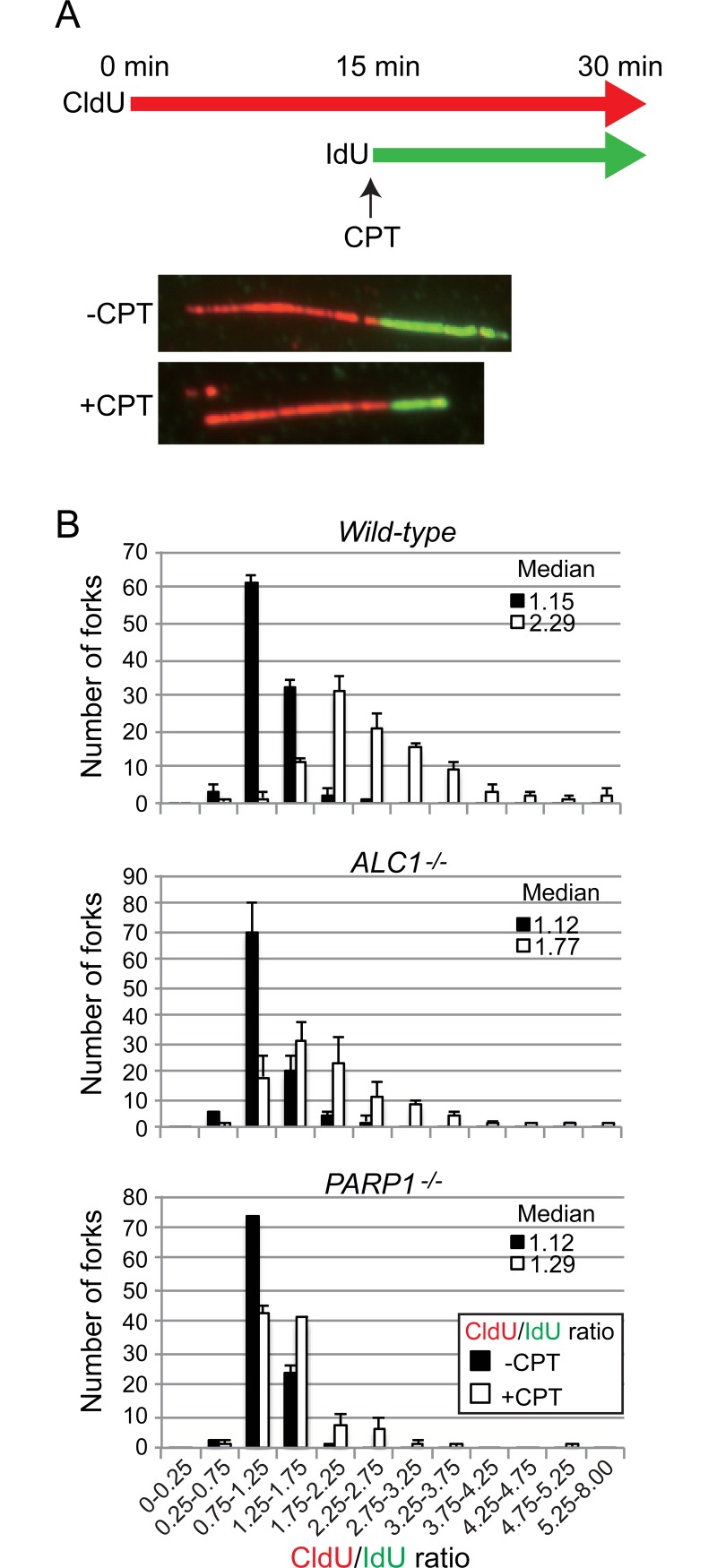
Role played by the ALC1-PARP1 pathway in safe fork-slowing following CPT exposure. (A) Representative images showing stained DNA fibers. DT40 cells were labeled sequentially with CldU and IdU with or without CPT treatment after CldU labeling. (B) Distribution of CldU/IdU ratios for replication forks in cells exposed to CPT. Indicated cells were incubated in medium containing CldU (25 μM) for 15 min, then incubated in medium containing IdU (250 μM) with CPT (10 μM) or without CPT for 15 min. The CldU/IdU ratios are shown on the x-axis. The number of fibers in each section is shown on the y-axis. 100 forks from each cell line were analyzed. Error bars represent standard deviations from three independent analyses.

Since CPT induces replication-fork slowing [21, 22, 37], we next investigated what role ALC1 might play in the regulation of replication forks. We measured the length of the replicated tract before (CldU) and after (IdU) CPT treatment in *ALC1*^*-/-*^, *PARP1*^*-/-*^, and *wild-type* cells, then compared the CldU/IdU ratios (Fig 5). Consistent with our previous study, replication tracts in the *wild-type* cells were significantly shortened following CPT exposure, with a median CldU/IdU ratio of 2.29± 0.15 (Fig 5). In *ALC1*^*-/-*^ cells, replication-fork slowing was partially impaired, with a median CldU/IdU ratio of 1.77± 0.08 (Fig 5). *PARP1*^*-/-*^ cells showed an even more pronounced defect in replication-fork slowing (median CldU/IdU ratio of 1.29± 0.01). These results indicate that ALC1 is involved in the regulation of replication-fork slowing. The critical role played by the PARP pathway in safe fork arrest [21, 22] and the data showing an epistatic relationship between *ALC1* and *PARP1* in CPT tolerance combine to suggest that ALC1 may collaborate with the PARP pathway in the regulation of replication forks at TopI-cc sites.

In this study, we uncovered a previously unappreciated function of ALC1 in the regulation of DNA replication, wherein ALC1 slows replication-fork progression at TOPI-cc under the PARP pathway and thereby prevents replication-fork collapse. This conclusion is supported by a DNA-fiber assay demonstrating that ALC1 slows the progression of replication forks after CPT treatment (Fig 5). These data unveil the role played by ALC1 in the regulation of replication-fork progression following DNA damage. ALC1 contributes to cellular tolerance to DNA damage through multiple mechanisms: promotion of base-excision-repair pathways and prevention of DNA replication-fork collapse following DNA damage to template strands [4, 11–13, 38].

**Fig 6:**
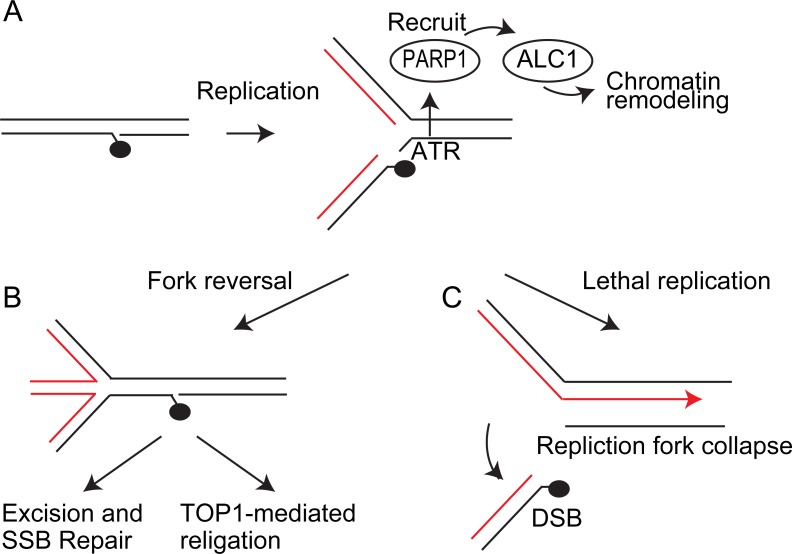
A model. Schematic representation of the model showing the regulation of safe replication fork arrest at TopI-cc by PARP-ALC1 pathway. (A) Replication-fork arrest at TOP1-cc activates the ATR-CHK1 checkpoint, which in turn activates the PARP1. PARylation via PARP1 enzyme mediates recruitment of ALC1. ALC1 mediates chromatin remodeling and facilitates replication-fork reversal. (B) As a result of fork reversal, TOP1-cc is located at the 3’ end of SSB and efficiently excised and repaired. (C) Lethal replication results in one-end DSB and following fork collapse.

An important question is, how does ALC1 contribute to the regulation of replication forks? It is possible that, during fork-slowing, some aspect of the PARP-ALC1 axis is under the control of the S-phase-checkpoint pathway that comprises ATR-CHK1 [39]. TopI-cc interferes with DNA replication by avoiding origin firing and fork progression via the ATR-CHK1 signal pathway [37]. One possible scenario is that transient replication-fork arrest at TOPI-cc sites activates the ATR-CHK1 checkpoint (Fig 6A), which in turn activates the PARP-ALC1 axis, leading to fork reversal in which the newly synthesized strands are annealed to one another (Fig 6B). This hypothesis is supported by previous reports that visualize fork reversal at TOPI-cc and other DNA damage sites on template strands using an electronic microscope [21, 40]. Fork reversal might also require homologous recombination mechanisms, including the RAD51-XRCC3 complex [41]. As a result of fork reversal for the safe replication fork slowing, TOP1-cc at the 3’ SSB end might be efficiently excised by nucleases including TDP1 (Fig 6B). In addition, removal of CPT allows TOP1-mediated religation, [14, 15] (Fig 6B). Loss of fork reversal and following lethal replication may result in one-end DSB at fork leading to fork collapse (Fig 6C).

The *ALC1* gene is frequently amplified and overexpressed in human hepatocellular carcinoma and numerous solid tumors. This overexpression is associated with lymph-node metastasis, tumor differentiation, and distant metastasis [42, 43], suggesting that the *ALC1* gene plays a role in invasion and metastasis of cancer cells. In contrast to the hypoxic microenvironment of solid tumors, malignant cells are exposed to a high concentration of oxygen during hematogenous metastasis. One possible explanation is that the overexpression of ALC1 might increase cellular tolerance to oxidative stress during metastasis. Further analysis of ALC1’s role might clarify the role of ALC1 in cancer development.
